# Molecular Characterization of Advanced-Stage Melanomas in Clinical Practice Using a Laboratory-Developed Next-Generation Sequencing Panel

**DOI:** 10.3390/diagnostics14080800

**Published:** 2024-04-11

**Authors:** Thais Maloberti, Antonio De Leo, Sara Coluccelli, Viviana Sanza, Elisa Gruppioni, Annalisa Altimari, Francesca Comito, Barbara Melotti, Paola Valeria Marchese, Emi Dika, Federico Venturi, Barbara Corti, Giulia Ciccimarra, Crina Adriana Ciceu, Giovanni Tallini, Dario de Biase

**Affiliations:** 1 Solid Tumor Molecular Pathology Laboratory, IRCCS Azienda Ospedaliero-Universitaria di Bologna, 40138 Bologna, Italy; thais.maloberti2@unibo.it (T.M.); antonio.deleo@unibo.it (A.D.L.); sara.coluccelli2@unibo.it (S.C.); v.sanza@ausl.bo.it (V.S.); elisa.gruppioni@aosp.bo.it (E.G.); annalisa.altimari@unibo.it (A.A.); giovanni.tallini@unibo.it (G.T.); 2 Department of Medical and Surgical Sciences (DIMEC), University of Bologna, 40138 Bologna, Italy; emi.dika3@unibo.it (E.D.); federico.venturi@hotmail.it (F.V.); 3 Oncology Unit, IRCCS Azienda Ospedaliero-Universitaria di Bologna, 40138 Bologna, Italybarbara.melotti@aosp.bo.it (B.M.); paola.marchese7@studio.unibo.it (P.V.M.); 4 Oncologic Dermatology Unit, IRCCS Azienda Ospedaliero-Universitaria di Bologna, 40138 Bologna, Italy; 5 Pathology Unit, IRCCS Azienda Ospedaliero-Universitaria di Bologna, 40138 Bologna, Italy; barbara.corti@aosp.bo.it; 6 Department of Pharmacy and Biotechnology, University of Bologna, 40138 Bologna, Italy; giulia.ciccimarra@studio.unibo.it (G.C.); crinaadriana.ciceu@studio.unibo.it (C.A.C.)

**Keywords:** melanoma, NGS, mutation, *BRAF*, *TERT*, sequencing

## Abstract

Cutaneous melanoma is one of the most lethal tumors among skin cancers, characterized by complex genetic and molecular alterations that result in uncontrolled cell proliferation and metastatic spread. Next-generation sequencing (NGS) enables the simultaneous examination of numerous genes, making this molecular technique essential for melanoma diagnosis, prognostic stratification, and therapy planning. Herein, we present the experience with our laboratory-designed NGS panel for the routine assessment of advanced-stage melanoma. A total of 260 specimens of advanced-stage melanomas were evaluated utilizing a laboratory-developed multi-gene NGS panel, which allowed the investigation of 229 amplicons in 25 oncogene/oncosuppressor genes. The NGS panel proved to be a reliable tool, failing to produce results in only 1.2% of the samples tested. *BRAF* and *TERT* were the two more commonly altered genes in 44.0% and 59.9% of samples, respectively. In 59.3% of the mutated cases, at least two concomitant variants were detected. In eight cases, both primary lesion and metastatic disease were analyzed by NGS. In all specimens (8/8, 100%), a perfect concordance in variants harbored by the primary and recurrence lesions was observed. Finally, this study described the validity of a laboratory-developed multi-gene NGS panel built specifically for advanced-stage melanomas in ordinary clinical practice.

## 1. Introduction

Cutaneous melanoma is one of the most lethal tumors among skin cancers, and its incidence is rising worldwide. It is caused by a complex interplay of genetic and epigenetic alterations that drive its initiation, development, and metastasis. These changes frequently impair essential signaling networks that regulate cell growth, proliferation, differentiation, and survival. The vast majority of melanomas are sporadic, and one of the more common molecular alterations is the mutation of the *BRAF* gene. 

*BRAF*. One of the most well-known genetic alterations in melanoma is the mutation of the *BRAF* gene, specifically the p.V600E mutation, which occurs in around 50% of cases. BRAF is a serine/threonine kinase protein involved in the MAP kinase pathway, which controls cell growth and proliferation. The most common variants are *BRAF* class I mutations (mainly BRAF p.V600E, followed by BRAF p.V600K), which are almost exclusively induced by the oncogenic/pathogenetic key role of UV radiation [1]. However, other non-V600 BRAF variants may be found in advanced-stage melanoma [1,2,3]. These variations in the *BRAF* gene may cause constitutive protein activation, which results in uncontrolled cell proliferation and tumor formation [3,4].

*TERT*. Telomerase reverse transcriptase (TERT) mutations are key players in melanoma genesis and progression. *TERT* activity is typically carefully regulated, but changes can disrupt this control, resulting in unregulated cell proliferation and cancer development. Recurrent mutations in the promoter of the *TERT* gene were initially detected in melanoma and then in various additional cancer types [5,6,7]. *TERT* promoter mutations are the most common type of *TERT* alterations in melanoma, accounting for up to 65% of cutaneous melanoma [1,8]. These mutations increase *TERT* expression, which helps cancer cells grow and survive. Mutations in the promoter region of *TERT* are related to reduced disease-free survival, increased tumor recurrence, and an increased rate of metastasis in melanoma [8,9,10,11].

*NRAS*. Mutations in the *NRAS* gene are found in about 15–20% of melanomas [1,12]. Hot-spot mutations in the *RAS* genes are generally at the Q61 codon and less frequently in G12 or G13 [1]. Mutations in *RAS* genes cause the constitutive activation of the MAPK pathway, resulting in increased cellular proliferation, survival, and resistance to apoptosis [13]. Inhibiting proteins farther down the RAS pathway, such as MEK and ERK, can indirectly prevent the carcinogenic signals triggered by *RAS* mutations [13].

Other common genetic alterations observed in melanoma are the loss of function of the *CDKN2A* tumor suppressor gene, *TP53* mutations/deletions (thus resulting in the loss of heterozygosis) but with a lower frequency and a lower magnitude effect compared to other solid tumors, and *KIT* mutations mainly detected in acral and mucosal melanoma [1,12,14,15,16].

Next-generation sequencing (NGS) enables the simultaneous sequencing of numerous genes with a very high depth of coverage. Given the ongoing discovery of molecules as potential targets or molecules that are accountable for treatment resistance mechanisms, using a classic single-gene approach is becoming challenging. The adoption of multi-gene panels is now essential for the molecular investigation of solid tumors, including melanoma. According to the ESMO guidelines, “*If the tumour is *BRAF* wild-type (WT) at the V600 locus (class I BRAF mutant), sequencing the loci of the other known minor *BRAF* mutations (class II and class III BRAF mutant) to confirm WT status and testing for *NRAS* and *c-kit* mutations are recommended [II, C] […]. Alternatively, a clinically validated next-generation sequencing panel covering all key oncogenic drivers is increasingly being carried out*” [17,18].

The present study aims to disclose a laboratory-designed multi-gene panel that allows for assessing advanced-stage melanoma in routine clinical practice.

## 2. Materials and Methods

A total of 260 cases of advanced-stage melanomas were analyzed for routine practice at the Molecular Pathology Laboratory of IRCCS Policilinico di S.Orsola in Bologna, Italy, from January 2021 to December 2023. All samples analyzed were extracted from formalin-fixed and paraffin-embedded (FFPE) histological blocks. Briefly, DNA was extracted from 2 to 3 10 μm thick sections, according to the selection performed by a pathologist on the last Hematoxylin and Eosin (H/E) slide. DNA was quantified using a Qubit fluorometer (Thermo Fisher Scientific, Waltham, MA, USA).

### NGS Analysis

The next-generation sequencing (NGS) analysis was performed using a multi-gene panel developed in the Molecular Pathology Laboratory of IRCCS Policlinico di S.Orsola [19]. The panel allows the analysis of the following hot-spot regions of 25 genes for a total of 229 amplicons (15.04 kb, human reference sequence hg19/GRCh37) in the following genes: *BRAF* (exons 11, 15), *CTNNB1* (exon 3), *EGFR* (exons 12, 18, 19, 20, 21), *EIF1AX* (exons 1, 2), *GNA11* (exons 4, 5), *GNAQ* (exons 4, 5), *GNAS* (exons 8, 9), *H3F3A* (exon 1), HRAS (exons 2, 3), *IDH1* (exon 4), *IDH2* (exon 4), *KIT* (exons 8, 9, 11, 13, 17), *KRAS* (exons 2, 3, 4), *MED12* (exons 1, 2), *MET* (exons 2, 14), *MYC* (exons 1-3), *NRAS* (exons 2, 3, 4), *PDGFRα* (exons 12, 14, 18), *PIK3CA* (exons 10, 21), *PTEN* (exon 5), *RET* (exons 5, 8, 10, 11, 13, 15, 16), *RNF43* (exons 2, 8), *SMAD4* (exons 6, 9, 10, 11, 12), *TERT* (promoter region, g.1295141–g.1295471), and *TP53* (exons 4, 5, 6, 7, 8, 9).

NGS was performed using the Gene Studio S5 Prime Sequencer (Thermo Fisher Scientific). Briefly, about 30ng of DNA was used per panel for the amplicon library preparation, performed with the AmpliSeq Plus Library Kit 2.0. In specimens where the pathologists highlighted the presence of abundant melanin, 3 μL of betaine 1N was added. Templates were prepared with an Ion Chef Machine and sequenced using an Ion 530 chip. Sequences were analyzed with the Ion Reporter tool (v. 5.18–Thermo Fisher Scientific). The filter chain query was applied as follows: 0.05 ≤ allele frequency ≤ 1.0. Filtered variants were then manually investigated. Only nucleotide variations detected in both strands and at least 5% of the total number of reads analyzed were considered for the mutational calls [19]. The pathogenicity of each mutation was assessed using the Varsome tool (https://varsome.com/, last access: 1 February 2024). Benign/likely benign variants were not considered in the present study. The research was approved by the local institution’s ethics committee. All data used in the present study were completely anonymized and aggregated.

## 3. Results

**Figure 1:**
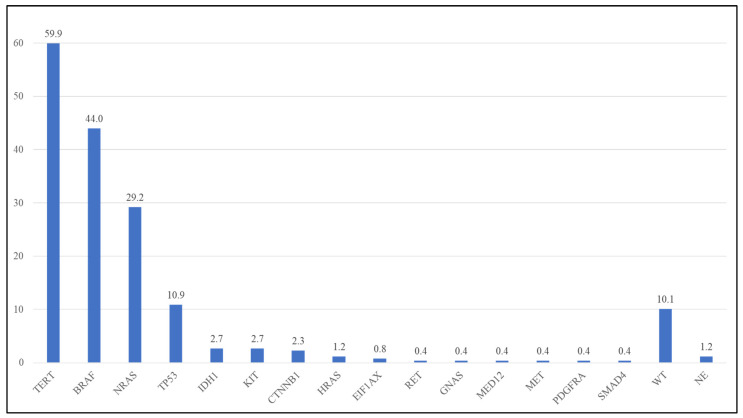
Frequency of mutations in the analyzed cohort. WT: wild type; NE: Not able to evaluate.

Of the 260 specimens analyzed by NGS, three (1.2%) were not evaluable due to low quality/quantity DNA. The following evaluations were then performed on a total of 257 specimens with evaluable NGS analysis. In 26 out of 257 specimens (10.1%), no alterations were detected in any of the analyzed genes. In the remnant 231 specimens, overall, 402 pathogenic/likely pathogenic/VUS (P/LP/VUS) variants were identified in 15 genes (Table 1, Figure 1, Supplementary Table S1). No P/LP/VUSs were detected in the remnant genes analyzed in the NGS panel. The *BRAF* gene and *TERT* promoter were the more altered markers in the analyzed cohort (Table 1, Figure 1, Supplementary Table S1).

*BRAF*. *BRAF* mutations were detected in 113 out of 257 specimens (44.0%). The *BRAF* p.V600E was the more frequent variant (82/113 *BRAF* mutated cases—72.6%), followed by p.V600K (21/113, 18.6%), and other rarer *BRAF* variants (overall 10/113, 8.8%—Table 2). The vast majority (107/113 mutated specimens—94.7%) of the *BRAF* variants were in exon 15, but in 6 cases (5.3% of BRAF mutated cases), *BRAF* variants in exon 11 were observed (Table 2, Supplementary Table S1). All variants were pathogenic or likely pathogenic. Three variants (p.V600) were *BRAF* class I mutations, two were *BRAF* class II, and 2 were *BRAF* class III variants (Table 2, Supplementary Table S1).

*NRAS*. The *NRAS* gene was mutated in 76/260 samples (29.6%). Almost all the *NRAS* variants (69/75 mutated cases—90.8%) were in exon 3, and the other ones (9.1%) were in exon 2 (Table 3). No variants were detected in *NRAS* exon 4. All *NRAS* variants were pathogenic or likely pathogenic. The p.Q61R was the more frequent variant (38/76–50.0% of the *NRAS* mutated cases), followed by the p.Q61K (24/76–31.6%) (Table 3, Supplementary Table S1).

*TERT*. The *TERT* promoter was mutated in 154/257 (59.9%) samples, resulting in the gene that was most frequently altered. The c.-146C>T (C250T) was found in 76/154 (49.4%) *TERT* mutated samples, the c.-124C>T (C228T) in 66 samples (42.9%), c.-138_-139delinsTT in 11 samples (7.1%), and c.-124_-125delinsTT in 1 (0.6%) specimen (Table 4, Supplementary Table S1). 

*TP53*. *TP53* variants were detected in 28/257 cases (10.9%). Mutations are detected in different exons (from 4 to 9) of the *TP53* genes and are almost different between them (Supplementary Table S1). One case (3.6%) had a variant in exon 4; six cases had a variant(21.4%) in exon 5; nine(32.1%) in exon 6; seven(25.0%) in exon 7; four(14.3%) in exon 8; and one(3.6%) in exon 9. All but one variant were P/LP mutations (Supplementary Table S1).

Other variants. Other variants were identified in a total of 34 cases (13.2%) in the following genes: *IDH1* (2.7%), *CTNNB1* (2.3%), *KIT* (2.3%), *HRAS* (1.2%), *EIF1AX* (0.8%), *RET* (0.8%), *GNAS* (0.4%), *MED12* (0.4%), *MET* (0.4%), *PDGFRA* (0.4%), and *SMAD4* (0.4%) (Supplementary Table S1). Interestingly, all the *IDH1*-detected variants (7 cases—100%) were p.R132C mutations instead of the more common p.R132H *IDH1* mutation (Supplementary Table S1).

### 3.1. Concomitant Mutations

In 137 of the 231 (59.3%) mutated cases, at least two concomitant variants were detected. *TERT* was the more common gene found to be mutated with other genes, found in 127 of 137 (92.7%) cases with concomitant mutations. Overall, in 82.5% of cases (127 of 154) harboring a *TERT* promoter variant, at least one other gene was mutated together with *TERT* (Table 5, Supplementary Table S2). The more frequent matching was between *TERT* and *BRAF*, 72 of 127 (56.7%) cases, followed by *NRAS* (39.4%), *TP53* (11.0%), and other genes (*IDH1*, *KIT*, *CTNNB1*—11.0% each) (Table 5, Supplementary Table S2). 

Of the 113 *BRAF*-mutated cases, 79 (69.9%) harbored at least one variant in another gene, mainly *TERT* (72 of 79 cases—91.1%). *NRAS* was mutated with other genes in 51 out of 76 (67.1%) mutated cases, mainly *TERT* (50 of 51–98.0%). In three cases, concomitant *BRAF*/*NRAS* mutations were observed, but in all these cases, the *BRAF* variant was a class III mutation, which is known to have low activity compared to the *BRAF* wild type (Supplementary Table S2).

Of the 28 cases with the *TP53* mutation, 22 (78.6%) had at least one other mutation and the combination *TP53*-*TERT* was detected in 12 of these 22 (54.5%) samples. Interestingly, all cases harboring *IDH1* (*n* = 7) or *CTNNB1* (*n* = 6) variants had a mutation in at least one other gene. As regards *IDH1*, 6 out of 7 mutations (85.7%) were concomitant with *TERT* variants, and all 6 mutated *CTNNB1* cases (100%) were also mutated in the *TERT* promoter.

### 3.2. Primary and Metastatic Lesions

In eight cases, both primary and metastatic lesions were analyzed by NGS. In all specimens, we detected a perfect concordance in variants harbored by the primary and recurrence lesions (Table 6). Three out of the eight samples harbored a *BRAF* p.Val600 variant together with a mutation in the *TERT* promoter region; three samples had an *NRAS* p.Glu61Arg mutation and a *TERT* promoter region mutation; one sample had a *BRAF* and *IDH1* mutation; and one case harbored four different variants in *BRAF*, *NRAS*, *TERT*, and *TP53* genes (Table 6). Intriguingly, all but one sample had a *TERT* promoter mutation concomitant with other pathogenic variants. All these specimens were considered only one time in the whole cohort of 260 cases.

## 4. Discussion

To date, the molecular characterization of metastatic melanomas for predictive purposes has primarily relied on the assessment of *BRAF* mutations for the use of BRAF inhibitors. However, the genetic changes that distinguish these cancers extend beyond the single BRAF p.V600 mutation.

From a technical standpoint, the evaluation of the *BRAF* mutation alone can be accomplished using “single-marker” approaches, such as real-time or pyrosequencing, which allow the mutation to be studied quickly and affordably. However, if one wants to characterize a larger number of molecular markers in addition to *BRAF*, such approaches become less cost-effective and are difficult to use in everyday practice due to the multiplicity of tests required for proper characterization. The advent of NGS in molecular diagnostics enabled us to integrate multigene analysis with great analytical sensitivity.

In recent years, a large number of multi-gene panels have become commercially available. However, these panels contain a large number of targets and are typically intended for specific tumors or genes. Creating custom/laboratory-developed multi-gene panels enables the selection of targets based on the demands of the medical community, as supplied by the molecular laboratory. These panels allow for the optimization of the number of specimens that can be evaluated in a single run, reducing expenses.

The use of multi-gene NGS panels enables the characterization of various genomic areas while maximizing time and costs. Furthermore, using lab-developed panels allows for the “design” of the panel to be based on clinical needs, incorporating markers such as *TERT* or *TP53* that may not be available in commercial “targeted” panels.

Although the ESMO guidelines for the use of NGS in patients with metastatic cancers do not include melanoma between the advanced neoplasms in which NGS is recommended, it is also true that these guidelines “*strongly recommends that clinical research centers perform multigene sequencing as part of their missions to accelerate cancer research and drug development through clinical trials, provide access to innovation to patients and to collect data*” [20]. Furthermore, the ESMO guidelines for the characterization of the diagnosis and treatment of melanomas suggest not to be limited to the single analysis of the *BRAF* V600 locus. In tumors that do not carry this type of mutation, the molecular analysis has to be extended not only to less common *BRAF* variants but also to other genes, such as *NRAS* and *KIT* [18]. The NGS multigene approach allows the simultaneous analysis of these potential hot spots, which is a preferable approach compared to sequential analysis (V600 WT → other *BRAF* variants not present → analysis of genes other than *BRAF*); also, in light of the data obtained in this study in which *BRAF* V600 mutations are commonly found together with *TERT* promoter variants, it suggests that non-class I *BRAF* mutations could coexist with variants in other driver genes.

The panel we created for the characterization of melanomas with gene alterations is consistent with what has been described in the literature. *TERT* was shown to be the most frequently mutant marker, particularly when combined with *BRAF*. The NGS panel was also demonstrated to be reliable, failing to produce results in only 1.2% of the samples tested.

Although *BRAF* is one of the most common mutations, our findings show that relying just on *BRAF* to characterize advanced melanomas is quite limiting. Identifying novel prognostic markers and therapeutic targets is greatly needed, as well as tailored characterization approaches, to detect patients at high risk of disease recurrence [21,22].

In addition to the aforementioned *TERT*, numerous mutations have been reported in *NRAS*, *TP53*, and *IDH1*, *KIT*, albeit at a lower frequency. Intriguingly, in the majority of analyzed samples (52.7%), more than one mutation was detected. The more frequent matching was between *TERT* and *BRAF* in 72 out of 127 analyzed samples (56.7%). The combination of *TERT* promoter mutations and BRAF p.V600E is expected to provide a strong genetic basis for tumor aggressiveness [23]. Furthermore, *TERT* mutations are being studied as possible therapeutic targets due to their role in melanoma progression. Strategies include developing drugs that directly inhibit the *TERT* function or target the mechanisms that induce *TERT* alterations [24].

*BRAF* and *NRAS* variants were confirmed to be mutually exclusive, except for *RAS* and *BRAF* class III variants. These latter variants are known to have low activity compared to the *BRAF* wild type and cannot directly phosphorylate MEK [3]. In fact, it has been previously described in the literature that *BRAF* class III variants may co-occur with *RAS*-activating mutations [17,25].

Interestingly, in all eight cases in which both primary and recurrence lesions were analyzed, a perfect match in the molecular status of the two specimens was observed, as reported in the literature [26,27]. Even if, in our cohort, the primary and metastatic samples showed the same molecular structure, the analysis of other molecular markers could be performed to understand whether variants that are not present in the primary lesion may be acquired in the metastasis.

In those cases that do not harbor any variants in the analyzed genes, other alterations may be present in markers other than those covered by the panel. In fact, although the data demonstrate that this panel was able to identify mutations in almost all advanced melanoma, in 10% of these, no alterations were identified. In these samples, it might be worth investigating other markers, such as using a panel for fusion genes (e.g., *ALK* fusions) [28] or a Comprehensive Genomic Profiling (CGP) panel [29,30], allowing the identification of mutations in uncommonly altered genes. With our panel, the primary and metastatic samples showed the same molecular structure; however, the extension to other markers could also be performed to understand whether there are any acquired variants in the metastases that are not present in the primary lesion.

We then provide the validation of a laboratory-developed, custom-designed multi-gene NGS panel. Using this laboratory-developed panel, we were able to analyze multiple types of cancers in the same run. This laboratory-developed NGS panel was designed to cover the diagnostic/prognostic/predictive clinical needs not only for melanomas but also for other tumors, such as CRCs (colorectal carcinomas), thyroid nodules, pancreatic lesions, gliomas, and GISTs (gastrointestinal stromal tumors) [19]. Because this panel is intended for the key gene targets of all the tumors stated above, it may be utilized to analyze several tumor types in a single run, reducing the turn-around time and NGS costs. In comparison to existing/commercial NGS multi-gene panels, the optimized selection of genes and the possibility of analyzing the relevant markers in different tumor types enables a high number of specimens to be analyzed in each run. This versatility is not possible with commercially available multi-gene panels that are dedicated to the in-depth analysis of specific tumors, whereas commercially available comprehensive multi-gene panels include a large number of targets, limiting the number of samples that can be analyzed in the same run. Pooling routine melanoma samples with other tumors enabled us to test 32–40 samples per run, with an average turnaround time of 7.1 working days. The cost of reagents was between EUR 200 and 250 per sample, depending on the number of specimens loaded in a given run, proving that an NGS-based approach may be less costly than a single-gene-based approach [31].

Considering that the analysis of other markers besides *BRAF* is recommended in melanoma [18], the multi-gene approach using an NGS technique is preferable to sequential testing [31,32]. Therefore, being able to test multiple genes in a single analysis allows for better molecular profiling of melanomas, and through this panel, it is possible to perform this while keeping costs relatively low and reporting times rapid.

In conclusion, this study describes the validation of a custom-designed multi-gene panel capable of analyzing relevant gene targets—25 oncogenes/oncosuppressor genes—in advanced-stage melanomas, which can be successfully used in routine clinical practice for prognostic/predictive clinical purposes.

## Figures and Tables

**Table 1:** Frequency of altered genes in the analyzed cohort and comparison with data obtained from TCGA.

Gene	Detected Frequency in Present Study (Lab-Developed NGS Panel) (*n* = 260)	Frequency in TCGA (Whole-Exome Sequencing) [1] *
*TERT*	59.9%	64.4% ^
*BRAF*	44.0%	51.2%
*NRAS*	29.2%	27.3%
*TP53*	10.9%	19.8%
*KIT*	2.7%	5.0%
*IDH1*	2.7%	4.1%
*CTNNB1*	2.3%	7.4%
*HRAS*	1.2%	NA
*EIF1AX*	0.8%	1.7%
*RET*	0.4%	6.6%
*GNAS*	0.4%	6.6%
*MED12*	0.4%	5.8%
*MET*	0.4%	6.6%
*PDGFRA*	0.4%	5.8%
*SMAD4*	0.4%	NA

* Only advanced-stage melanomas analyzed in the TCGA study, at *n* = 115, were considered for the comparison. ^ Performed by Sanger sequencing. NA: data not available.

**Table 2:** *BRAF* variants in the analyzed cohort.

*BRAF* Variant	Exon	Cases (%) *n* = 113	ACMG Classification	*BRAF* Class
p.V600E	15	82 (72.6)	P	I
p.V600K	15	21 (18.6)	P	I
p.V600R	15	3 (2.7)	P	I
p.K601E	15	1 (0.9)	LP	II
p.G466E	11	2 (1.8)	LP	III
p.S467L	11	3 (2.7)	P	III
p.G469A	11	1 (0.9)	P	II

ACMG: American College of Medical Genetics and Genomics; P: pathogenic; LP: likely pathogenic.

**Table 3:** *NRAS* variants in the analyzed cohort.

*NRAS* Variant	Exon	Cases (%) *n* = 76	ACMG Classification
p.Q61R	3	38 (50.0)	P
p.Q61K	3	24 (31.6)	P
p.Q61L	3	4 (5.3)	P
p.Q61H	3	2 (2.7)	P
p.G60E	3	1 (1.3)	P
p.G12D	2	2 (2.7)	P
p.G13R	2	1 (1.3)	P
p.G13S	2	1 (1.3)	P
p.G13V	2	1 (1.3)	P
p.Q22K	2	1 (1.3)	LP

ACMG: American College of Medical Genetics and Genomics; P: pathogenic; LP: likely pathogenic.

**Table 4:** *TERT* variants in the analyzed cohort.

*TERT* Variant	Cases (%) *n* = 154	ACMG Classification
c.-124C>T	66 (42.9)	P
c.-146C>T	76 (49.4)	LP
c.-138_-139delinsTT	11 (7.1)	LP
c.-124_-125delinsTT	1 (0.6)	LP

ACMG: American College of Medical Genetics and Genomics; P: pathogenic; LP: likely pathogenic.

**Table 5:** Genes more frequently found mutated in combination with other genes.

Gene	Cases out of Total Mutated Samples (%)	Gene More Commonly Mutated with
*BRAF*	79/113 (69.9)	*TERT* (72/79—91.1%)
*NRAS*	51/76 (67.1)	*TERT* (50/51—98.0%)
*TERT*	127/154 (82.5)	*BRAF* (72/127—56.7%)
*TP53*	24/28 (85.7)	*TERT* (14/24—58.3%)
*IDH1*	7/7 (100)	*TERT* (6/7—85.7%)
*CTNNB1*	6/6 (100)	*TERT* (6/6—100%)

**Table 6:** Cases in which both primary and metastatic lesions were analyzed.

#	Specimen	Variants
1	Primary lesion	*BRAF* p.Val600Glu *TERT* c.-124C>T
	Metastasis	*BRAF* p.Val600Glu *TERT* c.-124C>T
2	Primary lesion	*NRAS* p.Gln61Arg *TERT* c.-124C>
	Metastasis	*NRAS* p.Gln61Arg *TERT* c.-124C>T
3	Primary lesion	*BRAF* p.Val600Lys *IDH1* p.Arg132Cys
	Metastasis	*BRAF* p.Val600Lys *IDH1* p.Arg132Cys
4	Primary lesion	*BRAF* p.Ser467Leu *TERT* c.-146 C>T *NRAS* p.Gln61Arg *TP53* p.Ile195MetfsTer51
	Metastasis	*BRAF* p.Ser467Leu *TERT* c.-146 C>T *NRAS* p.Gln61Arg *TP53* p.Ile195MetfsTer51
5	Primary lesion	*NRAS* p.Gln61Arg *TERT* c.-146C>T
	Metastasis	*NRAS* p.Gln61Arg *TERT* c.-146C>T
6	Primary lesion	*NRAS* p.Gln61Arg *TERT* c.-146C>T
	Metastasis	*NRAS* p.Gln61Arg *TERT* c.-146C>T
7	Primary lesion	*BRAF* p.Val600Glu *TERT* c.-146C>T
	Metastasis	*BRAF* p.Val600Glu *TERT* c.-146C>T
8	Primary lesion	*BRAF* p.Val600Lys *TERT* c.-124C>T
	Metastasis	*BRAF* p.Val600Lys *TERT* c.-124C>T

## Data Availability

All data are included in the article/supplementary material.

## Supplementary Materials

The following supporting information can be downloaded at: https://www.mdpi.com/article/10.3390/diagnostics14080800/s1, Table S1: Details of variants detected in the analyzed cohort using the NGS panel. ACMG: American College of Medical Genetics and Genomics; P: pathogenic; LP: likely pathogenic; VUS: variant of uncertain significance; c.: coding reference sequence; Table S2: Concomitant mutations detected in the analyzed cohort.
